# Consecutive series of 226 journey bicruciate substituting total knee replacements: early complication and revision rates

**DOI:** 10.1186/1471-2474-15-395

**Published:** 2014-11-25

**Authors:** Bernhard Christen, Michal Neukamp, Emin Aghayev

**Affiliations:** Department of Orthopaedic Surgery, Salem Spital, Schänzlistrasse 39, 3000 Bern 25, Switzerland; Institute for Evaluative Research in Medicine, University of Bern, Stauffacherstrasse 78, 3014 Bern, Switzerland

**Keywords:** Journey, Total knee replacement, Navigation, Learning curve, Knee surgery complication, Knee surgery revision, Revision rate

## Abstract

**Background:**

The Journey bicruciate substituting (BCS) total knee replacement (TKR) is intended to improve knee kinematics by more closely approximating the surfaces of a normal knee. The purpose of this analysis was to address the safety of Journey BCS knees by studying early complication and revision rates in a consecutive case series.

**Methods:**

Between December 2006 and May 2011, a single surgeon implanted 226 Journey BCS total knee prostheses in 191 patients (124 women, 67 men) who were eligible for study. Mean age at surgery was 68 years (41–85 years).

Outcome measures were early complications and minor and major revision rates. All complications were considered, irrespective of whether conservative treatment or revision was required.

**Results:**

The average implantation time was 3.5 years (range 1.3-5.8 years). Thirty-three complications (14.6% of 226 knees) required minor or major revision surgery in 25 patients. The remaining eight patients were treated conservatively. Sixteen minor revisions were performed in 12 patients. Thirteen major revisions were required in 13 patients, which results in a rate of 1.65 major revisions per 100 component years. The linear trend of the early complication rate by treatment year was not significant (p = .22).

Multivariate logistic regression showed no significant predictors for the occurrence of a complication or for revision surgery. A tendency towards higher complication rates was observed in female patients, although it was not significant (p = .066).

**Conclusions:**

The complication and revision rates of the Journey BCS knee implant are high in comparison with those reported for other established total knee systems. Caution is advised when using this implant, particularly for less experienced knee surgeons.

**Electronic supplementary material:**

The online version of this article (doi:10.1186/1471-2474-15-395) contains supplementary material, which is available to authorized users.

## Background

Total knee replacement (TKR) can offer reasonable pain relief and good long-term results[1]. Nevertheless, up to 30% of the patients are for various reasons not satisfied with the outcome of TKR[2, 3]. One reason is persistent pain, which is mainly anterior and usually depends on activity[2, 4]. Further reasons for dissatisfaction include functional deficits in daily life and limitations in sport[2, 5]. Other, unfulfilled patient expectations are also important.

After TKR knees can be quite stable in the coronal and sagittal planes and show an acceptable range of motion, but most are far from normal[6–8]. This is often an obvious consequence of incomplete restoration of a normal knee situation[2]. The surfaces of the knee can be adequately reproduced, and replacement of menisci and cartilage by polyethylene (PE) seems an acceptable compromise. Although the medial and lateral collateral ligaments can be balanced sufficiently to provide stability, restoring or copying the functions of the cruciate ligaments remains a major problem[9, 10]. The lack of an anterior cruciate ligament (ACL) is more or less compensated for by the conformity of the PE liner. However, more difficulty is encountered when dealing with the posterior cruciate ligament (PCL)[9, 10]. In PCL resecting designs, stability is achieved by conformity of the PE (e.g., rotating platform), or a post-cam mechanism of so-called posterior stabilized (PS) knees. When leaving the PCL intact, as in cruciate retaining (CR) knees, the lack of correct PCL balancing leads to unpredictable pain and functional results. Different designs have different advantages and limitations, but all have, in varying degrees, abnormal and inconsistent kinematics, and none restores normal kinematics[3]. This explains the great variability in clinical outcome.

The Journey bicruciate substituting (BCS) TKR (Smith & Nephew, Memphis, USA)[11–14] is intended to improve knee kinematics by more closely approximating a normal knee with an asymmetric femoral component, the PE replicating 3° of tibial varus, and a medially concave and laterally slightly convex shape. The function of both the ACL and PCL is replicated by a post-cam mechanism that engages not only posteriorly but also anteriorly. Cam and post are asymmetrical to guide the femur in flexion to external rotation in relation to the tibia, and in full extension to an internal rotation known as the screw-home mechanism.

The goal of this knee system is to provide “guided motion,” which should lead to kinematics similar to the normal knee. This guided motion has been reported in different studies comparing the Journey BCS prosthesis to other TKR systems and normal knees[11, 12, 15]. Different in vivo fluoroscopic studies demonstrate that close to normal kinematic motions can be attained with the Journey knee prosthesis[7, 8, 15–18]. Nevertheless, despite its more closely resembling the normal knee than other implants patients with a Journey TKR still show a kinematic profile different from that of normal knees[12].

If guided motion is the right direction for the development of total knee replacement, the Journey BCS knee should lead to superior functional results and less pain than a conventional TKR without compromising safety, and these knees should have an increased longevity due to reduced wear and loosening rates.

The purpose of this analysis was to address the safety of Journey BCS knees by studying early complication and revision rates in a short term series of 226 consecutive TKR.

## Methods

Informed consent was obtained from all patients. Specific ethical approval was not required as this study was a retrospective analysis of anonymized data conducted under a blanket approval of such studies granted by the local ethics committee.

### Sample characteristics

Between December 2006 and May 2011, 208 consecutive patients underwent a TKR. Seventeen patients were excluded because of instability of the medial or lateral collateral ligaments greater than 10°. They were treated either with a rotatory hinge TKR (n = 13) or a constrained condylar knee TKR (n = 4).

In the remaining 191 patients, the Journey BCS total knee prosthesis (Smith & Nephew, Memphis, USA) was implanted in 226 knees by a single surgeon. 11.1% of these had a fixed varus (>3°) and 1.3% a fixed valgus (>3°) arthritis. Primary osteoarthritis was treated in 222 knees, rheumatoid arthritis in three, and post-traumatic arthritis in one. One hundred twenty-four patients were female (65%) and 67 male (35%). One hundred thirteen prostheses were implanted on the left side, and the same on the right. Mean age at surgery was 68 years (range 41 to 85 years).

Follow-ups were performed at 2, 4 and 12 months postoperatively, and then as required by complications. All follow-ups through August 31, 2012 were included in the study. The average follow-up intervals for the treatment years 2007, 2008, 2009, 2010, and 2011 were respectively 5.1, 4.2, 3.1, 2.3, and 1.5 years. All knees underwent radiographic imaging at baseline, early postoperatively, and 8 weeks and 1 year postoperatively. At baseline, both legs standing AP, long-leg standing AP, lateral, and skyline views were taken. Early postoperatively, long-leg standing AP, lateral in 90° of flexion, and skyline views were taken. At eight weeks and 1 year postoperatively, AP and lateral views under fluoroscopic control were performed, as well as a skyline view. Also at one year a standing AP view of both legs was repeated.

Outcome measures were early complications, and minor and major revision rates. A major revision was defined as an intervention replacing one or more parts of the original implant (femoral and/or tibial component, polyethylene, and patella button if resurfaced). The surgical learning curve was assessed by analysis of early complication and revision rates by treatment year. All complications were taken into account irrespective of whether conservative treatment or revision was required.

### Surgical technique

Mini-midvastus approach was used for 204 knees and lateral approach for 22 knees that included fixed valgus deformity and required an osteotomy of the tibial tuberosity. All the knees were operated with the tibial-cut-first and balanced gap technique. The first 102 TKR were performed using standard instrumentation with extramedullary tibial cut and intramedullary distal femoral cut alignment. For the next 124 TKR, CT-less navigation (PI Galileo, Smith & Nephew, Aarau, Switzerland) was used to define the tibial and distal femoral cuts. The main femoral cuts were performed with the balanced-gap technique in all 226 TKR in full extension and 90° of flexion. The knees were balanced in extension by an in- tube release on the concave side in case of fixed deformity before the distal femoral cut. The cut was performed as soon as an equal medial and lateral force applied by a tensioner (Smith & Nephew, Memphis, USA) led to a neutral mechanical axis. This was clinically controlled according to preoperative planning in the first group and by the navigation system in the second. The femoral rotation was defined using the tensioner, again in 90° of flexion, applying an equal force on the medial and lateral side. Then the femur was cut anteriorly and posteriorly parallel to the tibial cut, which is called the depending-cut technique. To minimize errors, the classical bony landmarks (transepicondylar and posterior condylar lines) were included as a safety check for the ligament balancing. The patella was mostly resurfaced with an inlay technique using reamers in appropriate sizes. All components were cemented in one process starting with the tibial component first, then the femoral one, and ending with the patella if resurfaced. With the prosthesis in place, the optimal PE thickness was determined using different trials. The system allows 1 mm increments with PE thicknesses of 9 to 13 mm and then provides 2 mm increments in thicker liners. The main focus in all knees was to reach a perfectly stable knee in extension, and in 30° and 90° of flexion on the medial side to guarantee optimal medial pivoting.

### Statistical analysis

The Cochran-Armitage test was applied for early complication and revision rates to analyze their trend over treatment years.

Multivariate logistic regression analysis was performed to find predictors for 1) occurrence of complication, and 2) minor and 3) major revision surgery. The following covariates were considered: patient age, gender (male/female), bilateral surgery (yes/no), femoral component size (2-5/6-9), tibial component size (2-4/5-8), patella replacement (yes/no), approach (mini-midvastus/other), and navigation (yes/no).

All statistical analyses were conducted using SAS 9.3 (SAS Institute Inc., Cary, NC) with an α = .05.

## Results

### Complications and revisions

The total implantation time of the 226 TKRs was 787.6 years, which results in an average of 3.5 years per knee (1.3-5.8 years). Thirty-three complications were observed (14.6% of 226 knees) and 25 patients required minor or major revision surgery (Tables 1,2 and3). A major revision, meaning an exchange of one or more prosthetic components, was performed in 13 patients, which gives a rate of 1.65 major revisions per 100 TKR years. Sixteen minor revisions were performed in 12 patients. Seven patients needed two revision surgeries, including four in whom minor revision was not successful and complete revision TKR was required. The remaining nine patients with a complication were treated conservatively (Table 1).

**Table 1:** **Complications and their treatment**

Complication	Patients	Conservative treatment	Minor revision	Major revision
Infection	2	-	3 arthroscopies incl. debridement	1 revision TKR
Pulmonary embolism	1	1	-	-
Death (not related to TKR)	1	-	-	-
Hematoma	1	-	1 arthroscopic evacuation	-
Skin necrosis	1	1	-	-
Periprosthetic femoral fracture	1	-	2 revision surgeries	-
Persistent pain without specific reason	1	-	-	1 revision TKR
Stiffness (flexion less than 90°)	5	2 closed mobilization	3 arthroscopic release and closed mobilization	1 PE exchange and secondary patella resurfacing 1 revision TKR after unsuccessful exchange of PE
Friction iliotibial band	6	1	5 arthroscopic release and debridement	-
Dislocation patella button	2	-	-	2 exchange of button
Patella fracture/ fragmentation	1	-	-	1 trabecular metal patella and new button
Dislocation of tibial tuberosity after cut	1	-	2 revision surgeries to fix multifragmentary tuberosity	-
Wrong side PE	1	-	-	1 exchange of PE
Midflexion instability	9	4	-	5 revision TKR, one after unsuccessful arthroscopic debridement
**Total**	**33**	**9**	**16**	**13**

**Table 2:** **Patients with midflexion instability**

Reason	Patients	Conservative treatment	Minor revision	Major revision
Internal rotation of femoral component and posterolateral instability	4	1	-	3 revision TKR
Dislocations of the primary unstable knees	(1)	-	-	(1 revision TKR)
Secondary instability	5	3	-	2 revision TKR after 1 unsuccessful arthroscopy
Dislocations of the secondary unstable knees	(3)	(2)	-	(1 revision TKR)
**Total**	**9**	**4**	**-**	**5**

Note: numbers in parentheses represent subgroups of the above mentioned patient group.

**Table 3:** **Comparison between the conventional and the navigation group**

	Conventional group		Navigation group		Total	
Number of knees	102		124		226	
Implantation time (years)	479.3		308.3		787.6	
Mean (years)	4.7		2.5		3.5	
	**Complication**	**Major revision**	**Complication**	**Major revision**	**Complication**	**Major revision**
Pulmonary embolism	1	-	-	-	1	-
Death	1	-	-	-	1	-
Skin necrosis	-	-	1	-	1	-
Periprosthetic fracture	1	-	-	-	1	-
Avulsion of tibial tuberosity	1	-	-	-	1	-
Hematoma	-	-	1	-	1	-
Infection	1	-	1	1	2	1
Chronic pain	1	1	-		1	1
Dislocation patella button	1	1	1	1	2	2
Fragmentation/necrosis of patella	-	-	1	1	1	1
Iliotibial band traction syndrome	3	-	3	-	6	-
Wrong side PE	-	-	1	1	1	1
Stiffness	4	2	1	-	5	2
Midflexion instability	4	2	5	3	9	5
**Total**	**18**	**6**	**15**	**7**	**33**	**13**

Serious general complications included a pulmonary embolism in one patient, which occurred during the primary hospital stay. One major hematoma had to be evacuated by arthroscopy during the first week after TKR. One patient died 4 months after surgery due to causes unrelated to the knee intervention.

Two of 191 patients had an infection, which results in a patient-based infection rate of 1.1%. One patient had an early infection caused by erysipelas, which was successfully treated by two arthroscopic debridements with no further treatment. The second patient complained about permanent pain after surgery and a stiff knee. An arthroscopic release and closed mobilization improved neither function nor pain. An infection was detected 21 months after surgery and was treated with a one-stage revision TKR. The first patient received penicillin for six weeks and the second patient a combination of chinolone and rifampicin for three months after revision surgery. Both patients recovered and there has been no recurrence of infection.

Two female patients suffered falls, each two weeks after TKR. One was treated for a supracondylar periprosthetic fracture with an interlocking plate. Failure of the proximal plate fixation required revision surgery by interlocking plating. The fracture healed uneventfully, though interference of the laterally prominent plate and the knee limited flexion to 90°. In the other, obese female patient, a screw fixation failure was documented after osteotomy of the tibial tuberosity with a lateral surgical approach. The fixation of the tibial tuberosity had to be revised twice.

One female patient had persistent tibial pain after TKR upon weight bearing. Although all examinations remained negative, the patient insisted on a revision TKR to increase tibial anchorage by a stem more than two years after primary surgery. Pain and walking capacity were unchanged after revision.

Five knees with flexion less than 90° were considered to be stiff. Two were mobilized under anesthesia. In one of these, no further treatment was necessary, while in the second exchange of the polyethylene by a thinner liner and secondary resurfacing of the patella solved the problem. The remaining three stiff knees were mobilized after arthroscopic release, with a near normal result in two and further revision of the third due to a chronic infection detected in the second year after surgery (see above).

Six patients, three in the conventional and three in the navigated group, complained about persistent lateral pain when moving or bearing weight on the knee, which is recognized as iliotibial band friction syndrome. Five of the six were released arthroscopically with reasonable success and one was treated conservatively. Prior local infiltrations did not improve the lateral pain.

One hundred sixty-four patellae of the 226 TKR were resurfaced and 62 were left alone. In all cases, resurfacing was done with an inlay technique using a reamer. Sixty-eight buttons were biconvex and 96 involved “classical” resurfacing with an undersurface plane and three pegs. All buttons were cemented. In one patient in whom resurfacing took place one year after TKR, for no detectable reason the patella developed a necrosis with loosening of the button two years after surgery. The patella was rebuilt using a trabecular metal core and a new cemented button. In two further cases the biconvex patella button dislocated 9 and 11 months postoperatively. Both knees were revised by converting to a resurfaced patella with three pegs. All three of these resurfaced patella complications involved biconvex patellae, which results in a biconvex patella complication rate of 4% and a dislocation rate of 3%.

In one right knee, a left polyethylene liner was inserted and had to be exchanged four months after surgery. This was detected at the first clinical and radiological control eight weeks postoperatively.

### Midflexion instability

Nine knees (4.0%) showed a symptomatic midflexion instability on the medial or lateral side, or both (Table 2). Four of these nine dislocated one or several times. In five patients with initially stable medial and lateral ligament situation in extension and 30° of flexion, the instability developed six or more months after TKR and was classified as secondary. The four primary cases, which were operated conventionally, showed an internal rotation of the femoral component of ≥5° in relation to the transepicondylar line in the CT scan. Because these cases additionally showed a midflexion instability on the lateral side they were considered to have an inadequate rotation of the femoral component. One patient refused further surgery and was treated with a “wait and see” approach. Three cases underwent a PS revision TKR with a standard PE liner under correction of the rotational alignment. One knee dislocated three times at four, five, and nine months after TKR, and was revised 10 months after the primary TKR.

Initially, five knees were judged to be stable at two- and four-month follow-up. For a friction syndrome of the iliotibial band, one knee was revised by arthroscopic debridement 13 months after TKR. In this knee, only 18 months after primary TKR a secondary medial and lateral midflexion instability was detected and required a revision TKR 24 months after the primary TKR. An overweight female patient with a longer lever arm also developed a secondary midflexion instability including a hyperextension one year after surgery. After a dislocation, which occurred 22 months after index surgery, her knee was revised by a constrained revision TKR. A fourth knee dislocated in deep flexion three times, 11, 39, and 52 months after surgery, and each time was treated by closed reduction. Finally, a farmer dislocated his knee twice when kneeling, four and seven months after TKR; it was reduced twice under anesthesia. After closed reduction, these last two patients were immediately allowed to bend the knee and to bear weight depending on pain and discomfort. The patients were instructed to strictly avoid varus stress in combination with a knee flexion of more than 30°. The patients have not required further revision and remain asymptomatic.

Increased midflexion instability was observed from the 50th TKR on, which led to two revision TKRs in the conventional group and three in the navigated group.

### The complication rate over time

**Figure 1:**
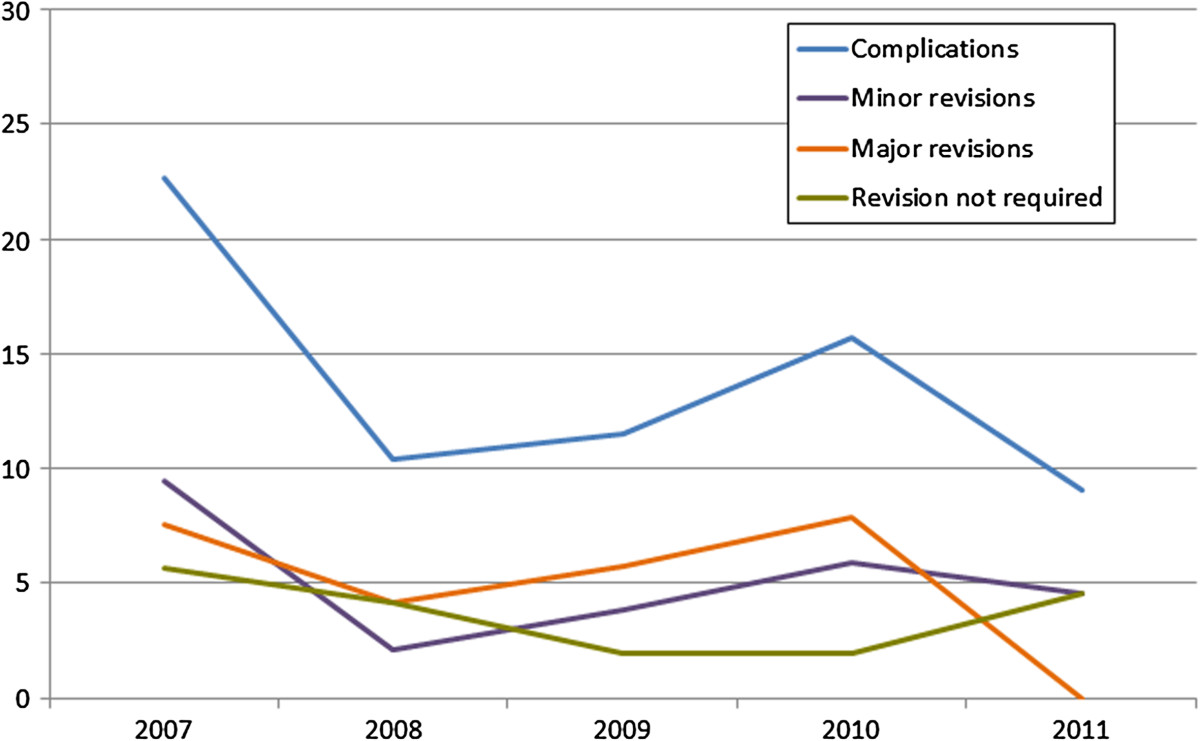
**Early complication, minor and major revision rates, and the proportion of patients with complications in whom no revision was required by the treatment year.**

Table 4 and Figure 1 show the decrease in early complication rates by the treatment year; the linear trend was not significant (p = .22). The complication rate during the first postoperative year was around 50% greater than the average rate of the five years studied.

**Table 4:** **Complication rate by the treatment year**

	Treatment year					
	2007	2008	2009	2010	2011	Total
Implantations	53	48	52	51	22	226
Complications	12	5	6	8	2	33
Complication rate	22.6%	10.4%	11.5%	15.7%	9.1%	14.6%

### Prediction of complication and revision surgery

The multivariate logistic regression showed no significant predictors for the occurrence of a complication or for revision surgery. Patient gender came closest to significance. Comparison of the higher complication rate in female patients (17.1%; 26/152 knees) to that of male patients (9.5%; 7/74 knees) had a p-value of .066. The rate of minor revisions was higher in female than in male patients, too (6.6% vs. 2.7%), though this difference also was not statistically significant.

Patients treated with the conventional surgical technique had higher unadjusted complication, and minor and major revision rates (17.5%, 18/103 knees; 6.8%, 7/103 knees; and 5.8%, 6/103 knees, respectively) than those treated with the navigated technique (12.2%, 15/123 knees; 4.1%, 5/123 knees; and 5.7%, 7/123 knees, respectively; Table 3). However, neither the difference in adjusted rates nor unadjusted rates was significant. The influences of other covariates on complication and revision prediction were far from significance (p > .12).

None of the joints with a complication had a varus or valgus axis alignment exceeding three degrees.

## Discussion

### Study results

Analysis of the 226 Journey prosthesis implantations shows an overall joint-based complication rate of 14.6%. Around one-third of the complications required a minor revision and another third a major revision.

The complication rate by treatment year suggests a surgical learning curve that was surmounted in the first year. One would expect the overall complication and revision rates to steadily decrease over time. However, a further increase of the overall rates took place in 2010 followed by an even steeper decrease and the lowest observed rate in the last year. However, the rates may change as the implantation time rises for this last cohort.

The rate of 1.65 major revisions per 100 observed component years documented in the study is high in comparison to the published rates for established knee systems. Schuh et al. have recently shown average TKR revision rates of 3.24% in European literature, 1.74% in US literature, and 3.33% in register-based studies[19], which correspond to 0.64, 0.16, and 0.51 revisions per 100 observed component years respectively[19]. The latest annual report of the National Joint Replacement Registry of the Australian Orthopaedic Association from 2012 showed 1.79 revisions (95% confidence limits 1.47-2.15) per 100 observed component years for the Journey prosthesis, which was the highest rate seen for a prosthesis with cement fixation[20]. Therefore, our hypothesis that the safety and reliability of Journey BCS knees does not differ from other, established TKR systems has to be rejected.

Although female patients showed generally higher complication and minor revision rates than male patients, in neither adjusted nor unadjusted comparisons were these differences statistically significant. The most frequent complication type was midflexion instability, followed by friction of the iliotibial band and stiff knee with a flexion less than 90°. Midflexion instability is often associated with revision surgery[21]. Even knees that have been carefully balanced in extension and at 90° flexion can exhibit midflexion instability during follow up; the exact mechanisms leading to the instability are not fully understood[22]. Neither the increased experience of the surgeon with the prosthesis in our study nor the aid of the CT-less computer navigation contributed to the reduction of the complication rate over time. All four patients with primary midflexion instability in our study were operated conventionally, yet navigation’s prevention of this complication is not obvious because navigation was used to control only the tibial and distal femoral cuts, not the rotational alignment of the femoral component. This last alignment was achieved in all cases using a tensioner under control of bony landmarks such as the posterior condylar line and the transepicondylar line. We suggest that the explanation for this complication occurring only in the conventional group in our study may be due to the surgeons’ learning curve—if it is not a coincidence. Midflexion instability could be attributed to the tibial-cut-first technique; it is accepted that this technique tends to raise the joint line. One might expect a clear difference between the non-navigated and navigated group, for in the latter the amount of resection can be determined more precisely. However, no difference was found in the multivariate regression analysis. Proper assessment of the effect of the navigation would require a randomized study design. The relative increase of the rate of midflexion instability from 50th TKR on was probably caused by the surgeon adding a little laxity after seeing some knees with a limited range of motion in too-tight implantations in the early phase. Adding a little laxity without perfect gap balancing in combination with the tibial-cut-first method may have caused or promoted a midflexion instability.

Surprisingly, in four knees femoral components showed inadequate internal rotation combined with posterolateral instability, which was judged as real malrotation despite using a gap balancing technique in all knees in 90° of flexion to define correct rotation. This error could be attributed to less invasive midvastus approaches for all knees except those with fixed valgus deformities. As the patella was never everted but only lateralized, the tension of the extensor apparatus may have limited the normal rotational alignment of the femur when spreading the tensioner with symmetrical force on the medial and lateral sides. This may cause limited and inadequate internal rotation of the femur. If the anterior and posterior cuts are performed parallel to the tibial cut, as was done in our case series, and if the femur is not properly positioned for the cut (e.g., restricted in rotation by the laterally displaced patella), the femoral cut of the lateral condyle might be larger than desired resulting in a less pronounced external rotation of the femoral component that is an inadequate internal rotation. By repositioning the extensor apparatus, the lateral ligaments would be too slack, resulting in posterolateral instability due to (too much internal) malrotation of the femoral component. In a guided motion knee such as the Journey BCS, this would lead to an exaggerated lateral roll forward and back. Combined with a smaller post with rounded tip, this could lead to dislocation. In addition to femoral internal malrotation, high flexion, a slight external rotation of the tibial component increasing the amount of lateral roll back in flexion, and a laxity of the lateral ligaments alone or in combination may be potential reasons for dislocations.

Luycks et al. documented the major revision rate of 2.0% after implantation of the Journey knee in his case series; while we observed a major revision rate of 5.7% in our study[14]. The author reports friction of the iliotibial band at a rate of 7.6%, which was 2.7% in our patients[14]. No particular pattern was associated with the friction of the iliotibial band in our study. This complication was associated neither with posterolateral instability nor with the dislocated knees. It appears that some of the knees suffered more from the extended lateral roll back of the Journey BCS knee than the others.

Zhou et al. reported good early postoperative results in 32 Journey prosthesis implantations. However, eight patients (25%) developed hydrarthrosis at 3–6 months after surgery, while two patients (6%) had a periprosthetic fracture and another two had an implant dislocation[13]. Arnout et al. reported four dislocations in a series of 1350 Journey BCS TKRs. The authors attributed the high dislocation rate (0.3%) to varus stress and a high degree of flexion attained in their patients[23]. In our study, dislocation of the patella button was responsible for two of the 13 major revisions. Because only the biconvex button showed this complication, in 2 of 68 cases (2.9%), while the resurfacing type had none in 96, use of the biconvex patella button should be discontinued. Failure of the biconvex patella design may be due to eccentric loading of the patella that results in a rocking horse effect it does not share with a classic button with a flat undersurface.

### Comparison of early complication and revision rates

The comparison of the complication and revision rates by the treatment year showed the highest rates in the first treatment year, which, as noted above, we believe is due to a surgical learning curve. In the following years the rates slightly decreased, with a small increase in 2010. For the patients treated in 2011 no major revision has yet been required. Since the follow-up time for the surgeries performed in this final year of the study is the shortest, the early revision rate of this year could increase.

### Conventional versus navigated implantation

Despite higher complication and revision rates in the conventional TKR compared to the navigated group, their differences are not significant. However, the shorter average duration of implantation for the navigated group (2.5 vs. 4.7 years) means an increase in the complication rate of the navigated group is possible. Recent systematic review of navigated versus conventional TKR by Zamora et al. showed comparable clinical outcomes between the two techniques with longer-term follow-ups suggesting that navigated TKR provided no benefit over conventional TKR in terms of functional improvement[24]. Recently, Baker et al. have analyzed a study cohort of 22,691 patients with a TKR and found no significant influence of computer navigation on early patient-reported Oxford knee scores and the EuroQoL-5D[25].

### Limitations

Although the differences in male and female early complication and minor revision rates were not statistically significant, as more Journey prostheses are implanted a larger sample size may change this picture making gender a significant covariate.

The study was based on a single surgeon’s experience. In our opinion, this is not a limitation of the study. On the contrary, we believe it has contributed to standardized study inclusion and exclusion criteria, operation technique, and complication and revision documentation. Further patient characteristics such as body mass index or level of physical activity may potentially influence the complication rate, but these unfortunately were not part of the documentation in the current study.

## Conclusions

We observed higher complication and revision rates for the Journey BCS knee implant than those reported for other established total knee systems. The use of navigation does not appear to significantly improve either the complication or the revision rates, though this is not a definitive conclusion. Caution is advised when using this implant, particularly for less experienced knee surgeons.

## Authors’ original submitted files for images

Below are the links to the authors’ original submitted files for images. Authors’ original file for figure 1

## Abbreviations

ACL: Anterior cruciate ligament
BCS: Bicruciate substituting
CS: Cruciate retaining
PCL: Posterior cruciate ligament
PE: Polyethylene
PS: Posterior stabilized
TKR: Total knee replacement.
