# Supplementary figures and images for: Consecutive series of 226 journey bicruciate substituting total knee replacements: early complication and revision rates
Source: BMC Musculoskelet Disord. 2014 Nov 25;15:395. doi: 10.1186/1471-2474-15-395 (PMC4289326; doi:10.1186/1471-2474-15-395)

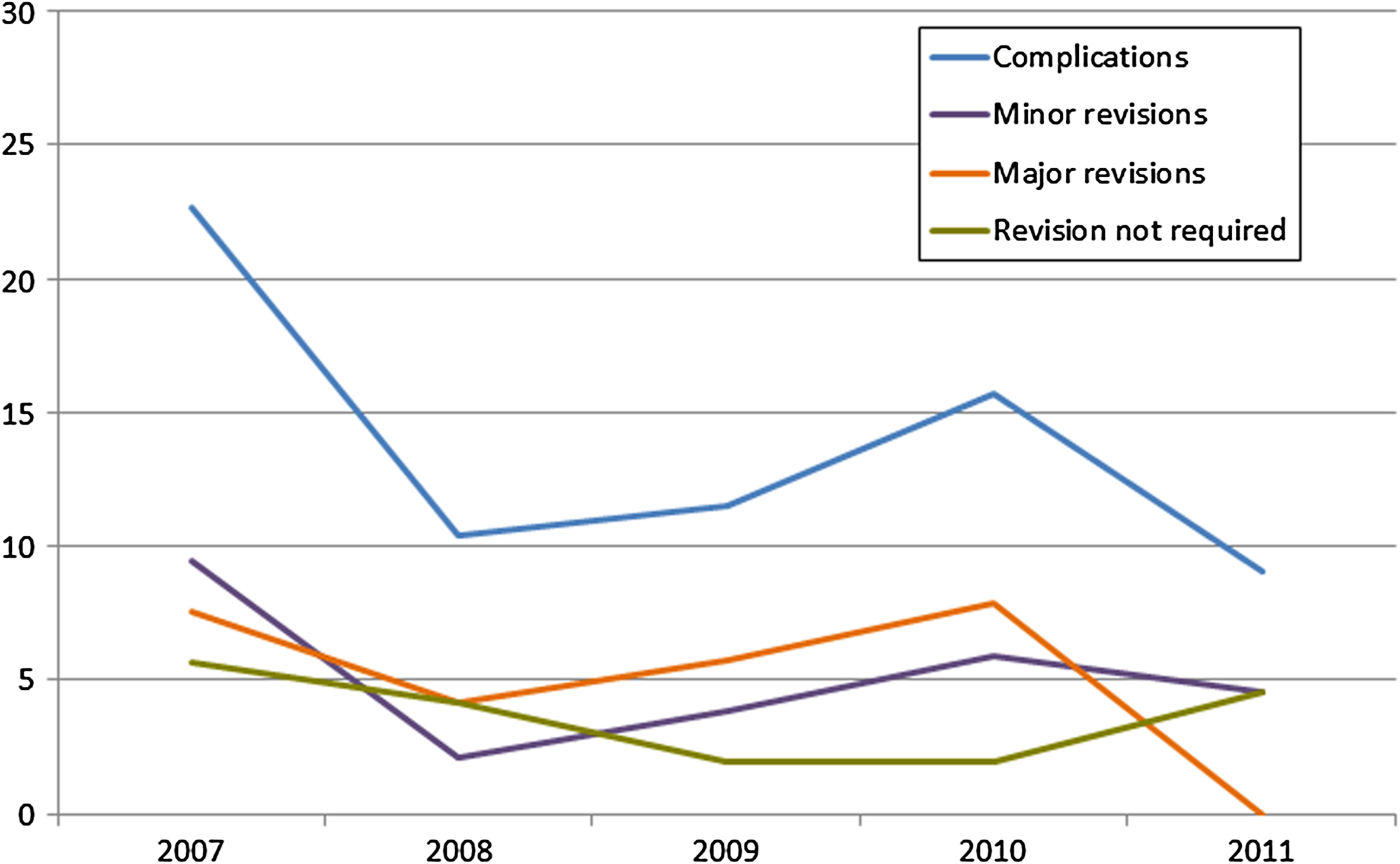

Supplement: Supplementary file 1 — Authors’ original file for figure 1 [file 12891_2014_2369_MOESM1_ESM.tif]
